# A sugar beet chlorophyll a/b binding protein promoter void of G-box like elements confers strong and leaf specific reporter gene expression in transgenic sugar beet

**DOI:** 10.1186/1472-6750-4-31

**Published:** 2004-12-05

**Authors:** Dietmar J Stahl, Dorothee U Kloos, Reinhard Hehl

**Affiliations:** 1 PLANTA Angewandte Pflanzengenetik und Biotechnologie GmbH, Grimsehlstr. 31, D-37555 Einbeck, Germany; 2 Institut für Genetik – Biozentrum, Technische Universität Braunschweig, Spielmannstr. 7, D-38106 Braunschweig, Germany; 3 Genedata GmbH, Lena-Christ Strasse 50, D-82152 Martinsried, Germany

## Abstract

**Background:**

Modification of leaf traits in sugar beet requires a strong leaf specific promoter. With such a promoter, expression in taproots can be avoided which may otherwise take away available energy resources for sugar accumulation.

**Results:**

Suppression Subtractive Hybridization (SSH) was utilized to generate an enriched and equalized cDNA library for leaf expressed genes from sugar beet. Fourteen cDNA fragments corresponding to thirteen different genes were isolated. Northern blot analysis indicates the desired tissue specificity of these genes. The promoters for two chlorophyll a/b binding protein genes (Bv*cab*11 and Bv*cab*12) were isolated, linked to reporter genes, and transformed into sugar beet using promoter reporter gene fusions. Transient and transgenic analysis indicate that both promoters direct leaf specific gene expression. A bioinformatic analysis revealed that the Bv*cab*11 promoter is void of G-box like regulatory elements with a palindromic ACGT core sequence. The data indicate that the presence of a G-box element is not a prerequisite for leaf specific and light induced gene expression in sugar beet.

**Conclusions:**

This work shows that SSH can be successfully employed for the identification and subsequent isolation of tissue specific sugar beet promoters. These promoters are shown to drive strong leaf specific gene expression in transgenic sugar beet. The application of these promoters for expressing resistance improving genes against foliar diseases is discussed.

## Background

Sugar beet (*Beta vulgaris *L.) is a biennial plant, a member of the *Chenopodiaceae *family [1]. In the first year after germination, a rosette of leaves develops while the taproot swells and accumulates sucrose. In the second year, flower initiation is induced after vernalization in the preceding winter. Beets are harvested at the end of the first year when sugar content of the taproot is high. Transgenic approaches towards modification of specific traits comprise the increase of pathogen resistance, the increase of sugar content and the improvement of sugar storage. These approaches require promoters that direct gene expression in a timely and spatial manner which is determined by the desired expression profile of the transgene.

In many cases improvement of transgenic traits in plants were achieved by using specific promoters [2,3]. Furthermore, to accomplish high tissue specific protein production in transgenic plants, often promoters from photosynthetic or storage specific genes are employed [4,5].

For the identification of desired promoters, a subtractive approach to enrich differentially expressed genes or a large scale approach to identify these genes in cDNA libraries may be employed prior to promoter isolation. One possible way to identify nonredundant clones in a cDNA library is the method of oligonucleotide fingerprinting (ofp) which was recently applied to sugar beet [6]. With this approach different cDNAs can be identified on a large scale basis within a cDNA library. While a large scale ofp approach is a feasible method to identify differentially expressed genes in different cDNA libraries, this method is very cost intensive and hence not applicable for many research groups.

A straight forward approach for the isolation of differentially expressed genes was achieved by the "Suppression Subtractive Hybridization" method (SSH) [7]. SSH is a cDNA- and a PCR-based technique that includes a step for the equalization of the abundance of different cDNA fragments during subtractive hybridization. Combined with suppression PCR, selective amplification of differentially expressed cDNA sequences was achieved without the application of physical separation methods [7]. This method was recently applied to isolate taproot expressed genes from sugar beet [8].

Here we have employed SSH for the isolation of leaf expressed sugar beet genes. Among the genes isolated was a cDNA fragment for a light-harvesting chlorophyll a/b binding protein (CAB). It is shown that sugar beet genotypes harbor either one or two *cab *genes that are both expressed. To investigate the use of the *cab *promoters for gene expression, the 5' regulatory sequences were isolated and linked to reporter genes. Transient reporter gene assays indicate that both promoters are expressed in sugar beet leaves. In transgenic sugar beet both promoters are expressed in green tissue. Sequence analysis revealed that the *cab*11 promoter, in contrast to the *cab*12 promoter, is void of G-box like regulatory sites with a palindromic ACGT core sequence. A leaf specific promoter in transgenic sugar beets can be employed for biotechnological applications.

## Results

### Identification of leaf expressed genes from sugar beet

To isolate cDNA fragments corresponding to leaf expressed genes, poly(A)+RNAs from leaf and taproot were isolated and subjected to cDNA synthesis. Suppression subtractive hybridization was performed as described in Methods. A total of 23 cDNA clones specific for the subtraction for leaf expressed genes were isolated and sequenced (data not shown). Fourteen out of 23 cDNAs were found to be different. Table 1 shows that 10 out of these 14 clones have homology mainly to nuclear encoded genes involved in the calvin cycle and in photosynthesis. Fragments L6 and L11 detect homology to the same gene but do not overlap. Four clones do not have any sequence homology to a known gene.

**Figure 1:**
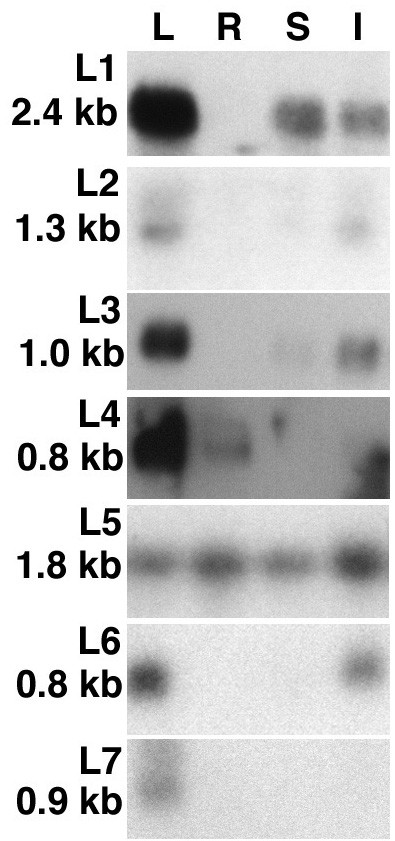
**Northern blot analysis with cDNA fragments enriched for leaf expressed genes**. Ten micrograms total RNA from leaves (L), taproots (R), stems (S), and inflorescences (I) were hybridized with the indicated cDNA fragments (Table 1). The sizes of the hybridizing transcripts are given in kilo base pairs (kb). The RNA gels were stained prior to blotting to confirm equal loading (data not shown).

To further confirm that the enriched cDNA fragments correspond to tissue specific genes, Northern blot hybridizations to total RNA isolated from leaves (L), taproots (R), stems (S), and inflorescences (I) were performed with 7 cDNA fragments. Figure 1 shows that all but one of the cDNA fragments hybridize specifically or much stronger to RNA from leaves than to RNA from taproots. Clone L5 hybridizes with similar intensity to RNA from all four tissues.

**Figure 2:**
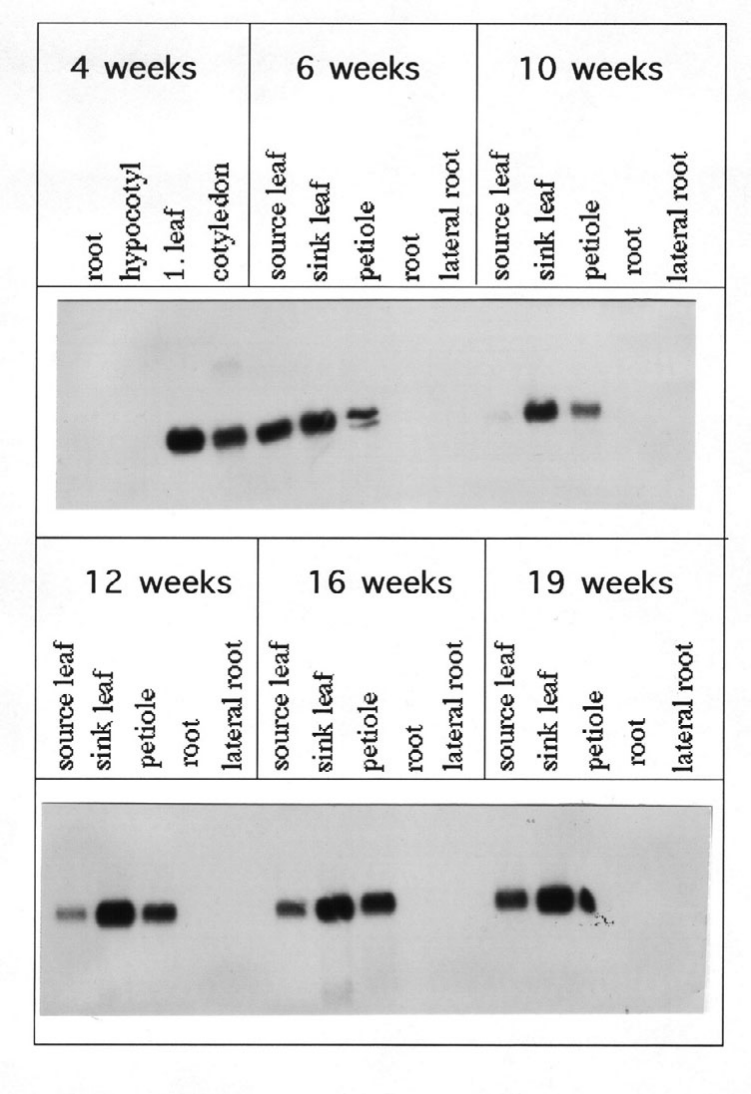
**Organ specific expression of the *cab *gene in the above ground parts of sugar beet during development**. RNA from root, lateral root, hypocotyl, cotyledon, petiole, sink and source leaf of sugar beets grown in the field was isolated 4, 6, 10, 12 16 and 19 weeks after sowing. Ten micrograms total RNA were separated in each lane and hybridized with the SSH fragment L2 (Table 1). The RNA gels were stained prior to blotting to confirm equal loading (data not shown).

The expression profile of the gene for SSH fragment L2 encoding a chlorophyll a/b binding protein (CAB) was analysed in more detail. RNA from different tissues and from different developmental stages were used in RNA blot experiments. RNA was isolated from different organs of sugar beets from a field 4, 6, 10, 12, 16 and 19 weeks after sowing. This interval covers the lifetime of a sugar beet in central Europe. Figure 2 shows that the gene is expressed only in above ground tissue like petiole, sink and source leaf regardless of the developmental stage. Since no expression is observed in below ground tissue like primary and lateral root the promoter of the *cab *gene was isolated and analysed with reporter gene fusions. Compared to the other genes, the expression profile of the gene for SSH fragment L2 showed the strongest tissue specificity during the different time points analysed (data not shown).

### Isolation of two promoters for the light-harvesting chlorophyll a/b binding protein

For the isolation of a sugar beet promoter corresponding to the *cab *gene, a complete cDNA clone was isolated (GenBank Acc. Nr. AJ579711, see Methods). A homology search with the encoded 252 amino acid long protein reveals a 87% identity to the *cab*11 and 85% identity to the *cab*12 gene from tomato encoding chlorophyll a/b binding protein [9].

**Figure 3:**
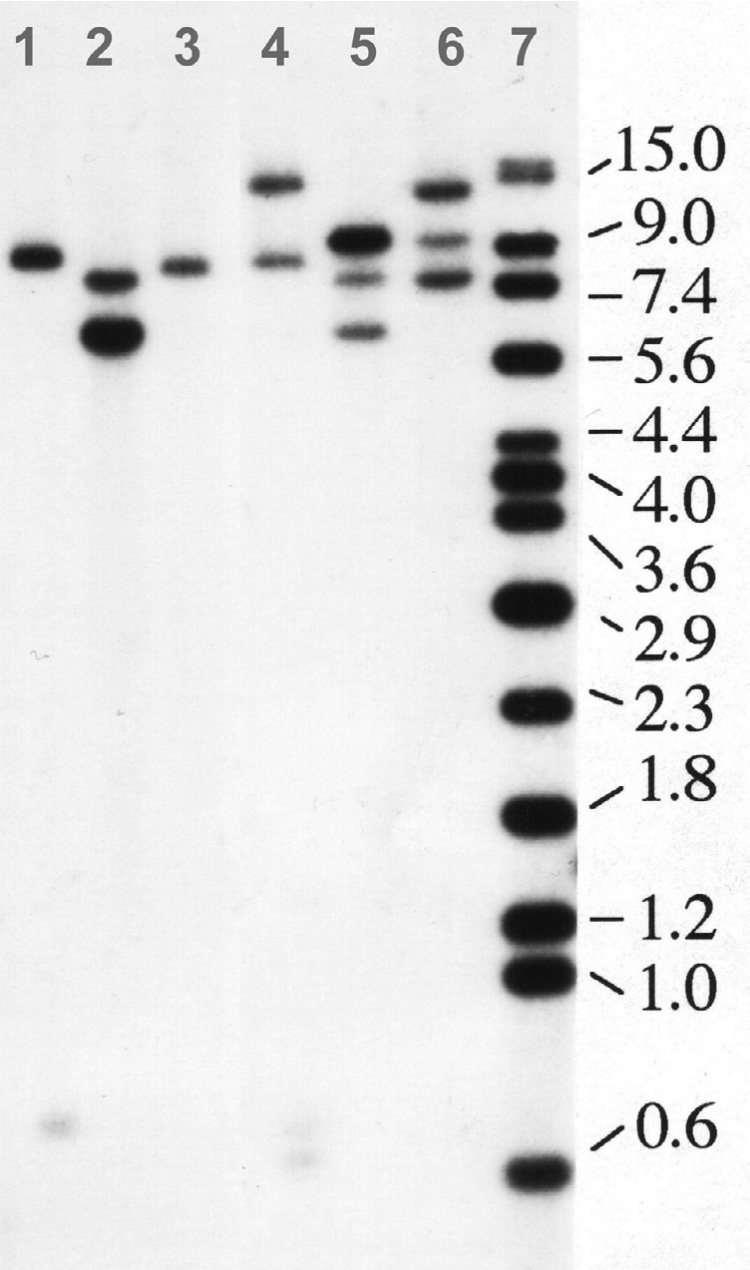
**The *cab *gene is encoded by one or two copies in different sugar beet genotypes**. Ten micrograms genomic DNA of genotype 4B5421 (lanes 1–3) and genotype 1K0088 (lanes 4–6) were restricted with different enzymes and hybridized with SSH fragment L2 (Table 1). Lane 1: *Pst*I, lane 2: *Hin*dIII, lane 3: *Eco*RI, lane 4: *Pst*I, lane 5: *Hin*dIII, lane 6: *Eco*RI, lane 7: size standard in kilo base pairs.

Prior to the isolation of genomic clones, a gene copy number analysis was performed. DNA from sugar beet genotypes 1K0088 and 4B5421 was digested with different restriction enzymes and hybridized to the L2 fragment (Table 1). Figure 3 shows that in genomic DNA from genotype 1K0088 three (*Eco*RI, *Hin*dIII) and four (*Pst*I) hybridizing fragments are detected, while only one (*Eco*RI) or two (*Pst*I, *Hin*dIII) fragments are detected in genotype 4B5421. From this result it is concluded that genotype 4B5421 harbors one and genotype 1K0088 harbors two copies of the *cab *gene.

Genomic clones for the two different genes were isolated (Methods). Sequence comparison between the cDNA and both genomic clones indicate a very high degree of sequence identity within the coding region. The CAB11 and CAB12 amino acid sequence differ only in one position (data not shown).

From both genes the promoter regions were subcloned into plasmid vectors and sequenced (Methods). Sequence of 1148 and 3049 base pairs, respectively, containing most of the upstream region were deposited to GenBank (Acc. Nr. AX449166 and AX449167). The 1148 bp promoter fragment is designated *cab*11 promoter and the 3049 bp fragment *cab*12 promoter. Both fragments harbor 51 base pairs coding region of the CAB protein and 113 (*cab*11) and 70 (*cab*12) base pairs upstream untranslated sequence. Upstream of the untranslated region only about 300 bp are homologous between the two promoters while the rest of the sequence is highly divergent (data not shown). Because the cDNA clone isolated before originates from the genotype 4B5421 and corresponds to the *cab*11 gene, it was investigated if the second gene is also transcribed. Towards these ends 5' RACE amplifications were performed with RNA from genotype 1K0088 and sequenced. This analysis revealed that the *cab*12 gene is also transcribed (data not shown).

### Transient expression assays in sugar beet leaves

To test whether the isolated promoters confer reporter gene expression in sugar beet leaves, a transient assay was performed. Towards these ends translational fusions of promoter fragments with the luciferase gene from *Photinus pyralis *in the vector pGEM-luc were constructed (see Methods). Table 2 summarizes the relative gene expression strength obtained with the different promoter reporter gene constructs when transformed into sugar beet leaves. Relative to the CaMV 35S promoter, the strength of all promoter fragments is approximately 20% and no significant drop in gene expression is observed between the largest and smallest promoter fragments. In summary, it can be concluded that the 1097 bp long fragment from the *cab*11 gene and the 342 bp fragment from the *cab*12 gene harbor all *cis*-regulatory sequences required for gene expression in sugar beet leaves at least under the conditions of the transient bioassay.

### The promoter of two chlorophyll a/b binding protein genes confers leaf specific and light inducible gene expression in transgenic sugar beet plants

**Figure 4:**
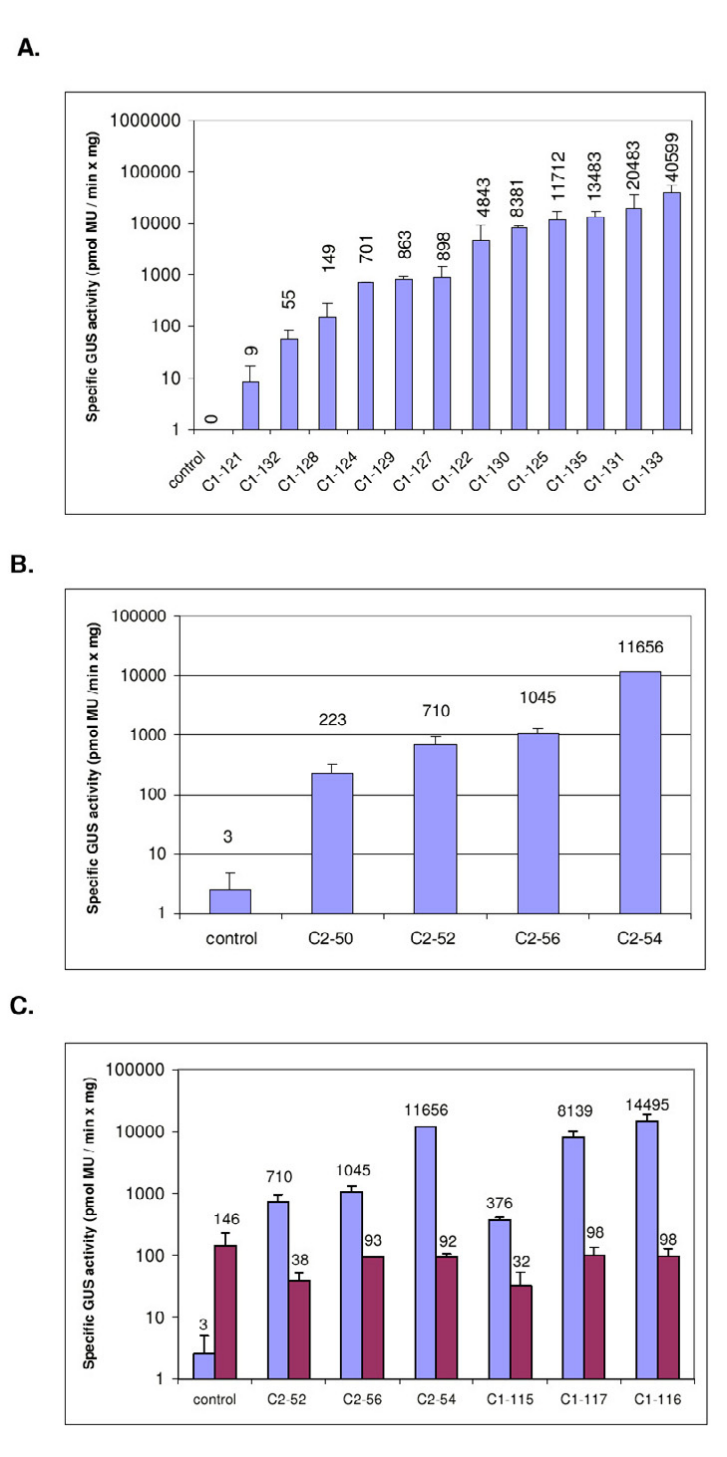
**GUS activity of *cab*11 and *cab*12 promoter reporter gene constructs in leaves and roots of transgenic sugar beets**. GUS activity was assayed using total protein prepared from the leaf or root. Each bar represents the activity of an individual transformant. The data are presented as the mean of three individual plants per transformant. The error bar describes the standard deviation of the mean. A. Activity of the *cab*11 promoter in leaves of transgenic sugar beet plants. B. Activity of the *cab*12 promoter in leaves of transgenic sugar beet plants. C. Comparison of the activity of the *cab*11 and *cab*12 promoter in leaves and roots of sugar beet. The GUS activity of the leaf extracts is shown by the left column and the activity of the root extracts by the right column.

To investigate if the *cab*11 and *cab*12 promoters from sugar beet drive reporter gene expression in transgenic plants, promoter reporter gene fusions were introduced via *Agrobacterium *mediated transformation in sugar beet (see Methods). The length of the *cab*11 promoter fragment is 1097 bp and the length of the *cab*12 promoter fragment 2998 bp. Leaves of transgenic lines transferred to the greenhouse were analysed for reporter gene activity. The *cab*11 promoter of 12 independent transformants showed a specific β-glucuronidase (GUS) activity from 9 to 40599 pmol Mu × min^-1 ^× mg^-1^, respectively (Fig. 4A). The *cab*12 promoter of 4 transformants showed a specific activity from 223 to 11656 pmol Mu × min^-1 ^× mg^-1^, respectively (Fig. 4B). These results indicate that the 1097 bp *cab*11 promoter fragment and the 2998 bp *cab*12 promoter fragment are sufficient to confer promoter activity to transgenic sugar beet leaves. Furthermore, the *cab*11 promoter seems to be stronger than the *cab*12 promoter although more transgenic lines were analysed for *cab*11 than for *cab*12.

In order to analyse if the *cab*11 and *cab*12 promoters confer tissue specific expression to sugar beet, the roots of three transgenic *cab*11 and three *cab*12 promoter lines were analysed. According to the strength of the *cab*11 and *cab*12 promoters in leaves (Fig. 4A and 4B) transgenic lines were selected which show low, moderate or high GUS activity in leaves. None of the lines showed GUS activity in the roots which was above the background level of nontransgenic control plants (Fig. 4C). Therefore the promoter activity of the *cab*11 and *cab*12 regulatory element is restricted to the above ground tissue of sugar beet and absent in roots. This result is consistent with the observations that transcripts of the *cab *genes are not detectable in the below ground tissue by Northern blot hybridization (Fig. 2).

To analyse *cab*11 and *cab*12 gene regulation in response to light, the reporter gene activtity of etiolated and green transgenic sugar beets was compared. *In vitro *shoots of the transgenic lines C1-121, C1-122, C2-50 and C2-52 were etiolated for 40 days in the dark. The GUS activity of the etiolated leaflets of one half of the plants was determined. Seven days after illumination the GUS activity of the remaining plants was determined after greening of the leaves. In two independent experiments the *cab*11 and *cab*12 promoter plants showed a 4,3 to 8,3 fold and a 95 to 118 fold induction during greening, respectively (Table 3). Although the GUS activity of the etiolated plants was comparable in the two assays, the greening plants showed a much stronger reporter gene activity in the second experiment. Apparently, different time points during the differentiation of etioplasts to chloroplasts were analysed. Time after illumination seems to have a strong influence on the level of promoter induction. Furthermore, gene expression is much stronger in these experiments compared to the analysis in transgenic plants (Table 3, Fig. 4). Finally, these results show that the *cab*11 and *cab*12 promoter are activated during the light induced plastid development.

### The *cab*11 promoter lacks G-box elements with a palindromic ACGT core sequence

**Figure 5:**
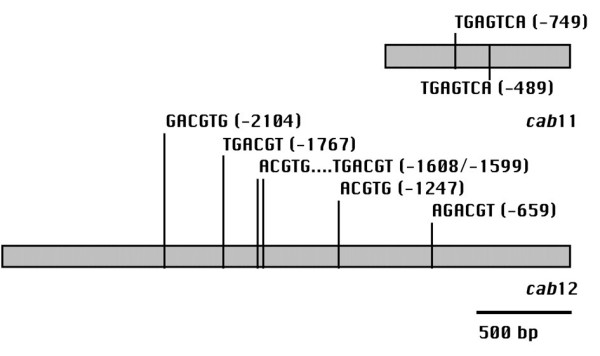
**Schematic representation of the *cab*11 and *cab *12 promoters with putative G-box elements**. Positions of putative G-box like elements are given relative to the translation start site in the *cab*11 and *cab*12 promoters. All binding sites except the single one at position -489 in the *cab*11 promoter are detected in the upper strand.

Gene expression is mainly regulated by the binding of transcription factors to specific *cis*-regulatory elements. Because the two sugar beet promoters show a similar expression profile, it was investigated if there are any differences or similarities in the composition of *cis*-regulatory sequences in both promoters. Towards these ends, a database-assisted approach was employed [10]. The Patch™ program [11] was used to inspect both promoter sequences for the occurence of plant transcription factor (TF) binding sites that are annotated to the TRANSFAC^® ^database [11]. The results reveal a large number of putative TF binding sites in both promoters (data not shown). Upon closer inspection of the results it was striking that the *cab*11 promoter, in contrast to the *cab*12 promoter was completely void of putative G-box like binding sites that contain a conserved ACGT core sequence and are recognized by bZIP transcription factors. Using the program Patch™ and entering a lower score boundary of 100% to detect only experimentally verified binding sites, only two putative binding sites for bZIP transcription factors were found in the *cab*11 promoter. Figure 5 shows the positions of these two motifs that both lack the ACGT core sequence. The sequence motifs at position -749 and -489 relative to the translation start site were found in the rice glutelin-B1 promoter and are bound by the rice bZIP transcription factor family RISBZ [12]. Both sites were also found to be bound by the tobacco bZIP transcription factor TGA1a in a pea lectin promoter [13].

Inspecting the sequence of the *cab*12 promoter for the ACGT core sequence of bZIP factor binding sites reveals 12 positions for this motif (data not shown). Using the program Patch™ six experimentally verified binding sites for bZIP factors were detected among these twelve sites that harbor the ACGT core in the *cab*12 promoter (Fig. 5). The motif at position -2104 is also present in the glutathione-S transferase 6 gene promoter of *Arabidopsis *where it is bound by the factor OBF4 [14]. The same site and the sites at position -1608 and -1247 occur in the embryonic abundant protein 1 promoter of rice and are recognized by the factors OSBZ8 and TRAB1 [15-17]. The sites at position -1767 and -1599 were recognized as bZIP binding sites in many other systems. The sequence TGACGT is part of the as-1 element of the CaMV 35S promoter that was shown to be bound by tobacco TGA1a, TGA1b, and TGA2.2 [18,19]. The site at position -659 is also present in the CaMV 35S promoter where it was shown to be bound by the wheat nuclear factor HBP-1 [20].

The observation that the *cab*11 promoter lacks G-box like elements with a conserved ACGT core sequence indicates that such sites are not required for leaf specific gene expression.

## Discussion

### The chlorophyll a/b binding proteins CAB11 and CAB12 from sugar beet belong to the light harvesting complex I – 730 (LHCI-730)

Subtractive hybridization was used to isolate leaf expressed genes from sugar beet. The goal was the identification of a promoter that drives leaf specific gene expression in transgenic sugar beet plants. Among seven analysed genes a cDNA fragment corresponding to a chlorophyll a/b binding protein gene was shown by RNA gel blot hybridization to be highly specific for green tissue (Fig. 1 and 2). Genomic DNA blot hybridizations indicate that the two sugar beet genotypes investigated harbor either one or two copies of the gene designated Bv*cab*11 and Bv*cab*12 (Fig. 3). A complete cDNA for the gene from genotype 4B5421 was isolated and encodes a protein of 252 amino acids that shows the highest homology (87%) to the *cab*11 gene from the light harvesting complex I (LHCI) in tomato [9]. This and homologies to other LHCI proteins indicate that the sugar beet gene belongs to the type IV LHCI complex [21]. Further support for this classification comes from the observation that the intron positions between *cab*11 from tomato and Bv*cab*11 from sugar beet are identical (data not shown).

LHCI can be subdivided into at least two different chlorophyll-protein complexes, one of which appears to be responsible for the 730 nm fluorescence of PSI (LHCI-730) and the other complex (LHCI-680) fluoresces at lower wavelength [21]. In barley the LHCI-730 complex was isolated as a heterodimer composed of the type I and type IV polypeptides [22]. Furthermore, tomato type I and type IV LHCI polypeptides (Lhca1/*cab*6a and Lhca4/*cab*11) expressed in *E. coli *form a heterodimer *in vitro *that closely resembles the native LHCI-730 dimer from tomato leaves [23]. Therefore, the sugar beet CAB11 and CAB12 proteins may be part of the LHCI-730 complex.

### G-box like elements are not a prerequisite for leaf specific gene expression

The promoters for both sugar beet *cab *genes were isolated and linked to reporter genes. Transient gene expression studies in sugar beet indicated that 1097 bp upstream of the ATG from the Bv*cab*11 gene and 342 bp upstream of the ATG from the Bv*cab*12 gene are sufficient for leaf specific gene expression in sugar beet (Table 2).

Promoter reporter gene constructs for Bv*cab*11 and Bv*cab*12 were stably transformed into sugar beet (*Beta vulgaris*, var. VRB). In sugar beet both promoters are expressed in leaves (Fig. 4).

When the promoter sequences of both *cab *genes where analysed for putative transcription factor binding sites, a striking difference was observed. The Bv*cab*11 promoter lacks G-box like sequences with a palindromic ACGT core. Are G-boxes required for light or leaf specific gene expression? A 268 bp fragment of the wheat *cab*-1 promoter functions as a light responsive and organ specific enhancer in transgenic tobacco [24]. Most notably the three regions that interact with nuclear factors and that were able to enhance gene expression of a 90 bp CaMV 35S minimal promoter did not contain a G-box sequence [24]. The requirement of G-box sequences for light specific gene expression has also been analysed directly [25]. A trimer of the G-box motif found in the spinach ribulose-1,5-bisphosphate carboxylase small subunit-1 promoter was fused to a 90 bp CaMV 35S minimal promoter. While a mutant of this G-box did not confer gene expression to the minimal promoter in the dark and under different light conditions, the G-box increased reporter gene expression under these conditions [25]. Reporter gene expression in the dark was comparably higher than under different light conditions. This is similar to the finding that a G-box like sequence in the *cab*1R gene of rice is necessary for high level transient expression of a reporter gene in tobacco leaf tissue [26]. Taken together, this indicates that the presence of G-box sequences may have a quantitative effect but may not be a prerequisite for green tissue specific gene expression in sugar beet.

### Biotechnological applications of leaf specific promoters in sugar beet

The major goal of this work was the isolation of a strong leaf specific sugar beet promoter that can be used for biotechnological applications. Disease control is one of the most important goals for biotechnological approaches towards improving sugar beet performance. There are many leaf spot diseases that are detrimental to the plant. For example, *Cercospora *leaf spot is one of the most widespread and destructive foliar diseases of sugar beet [27]. Expressing resistance improving genes in a strong and specific manner against pathogens causing foliar diseases may require a strong leaf specific promoter. With such a promoter, expression in taproots can be avoided which may otherwise take away available energy resources for sugar accumulation.

The work here shows that two promoters, Bv*cab*11 and Bv*cab*12, have been isolated that drive highly leaf specific gene expression in sugar beet (Fig. 4). No expression above background levels was detected for both promoters in sugar beet roots (Fig. 4C).

Based on the expression strength in transgenic plants, the Bv*cab*11 promoter may be suitable for biotechnological applications because it achieves a reporter gene activity comparable to the strong CaMV 35S promoter. CaMV 35S-mediated GUS activities in transgenic tobacco plants were reported as 113000 U (average of 10 plants, [28]) and 9000 U (average of 15 plants, [29]) in which 1 Unit refers to pmol 4-Mu produced min^-1 ^× mg protein^-1 ^[30].

The highest level of *cab*11 derived GUS expression is 40599 pmol Mu x min^-1 ^× mg^-1 ^which is comparable with the expression strength of the strong CaMV 35S promoter in tobacco.

## Conclusions

In summary, this work presents the isolation and expression analysis of two *cab *promoters from sugar beet. Particularly, the Bv*cab*11 promoter may be useful to drive strong and specific gene expression in transgenic host plants. The lack of bZIP binding sites harboring the ACGT core sequence could also be advantageous for transient analysis of bZIP transcription factors when using a Bv*cab*11 reporter gene construct as a transformation control. Furthermore these promoters may be useful to express resistance improving genes against foliar diseases such as *Cercospora *leaf spot.

## Methods

### Preparation of RNA and genomic DNA

Two different methods for RNA preparation were employed. To isolate RNA for cDNA subtraction, the procedure described below was followed. For some of the Northern blot analyses a method described earlier was employed [31].

For RNA isolation plant material was homogenized in liquid nitrogen and resuspended in a solution containing 4 M guanidinthiocyanat, 25 mM Tris-HCl, pH8 und 100 mM β-mercaptoethanol. After centrifugation (4°C, 10 min. at 3300 rcf) nucleic acids in the supernatant were precipitated by addition of 0.03 volume sodium acetate (3 M, pH5) and 0.75 volume ethanol (100%) and incubation over night at -20°C. After centrifugation (4°C, 10000 *g*, 10 min.) the nucleic acid containing pellet was dissolved in 20 ml 100 mM NaCl, 10 mM EDTA pH8, 50 mM Tris-HCl pH8, and 0.2% SDS. Afterwards, a phenol:chloroform (1:1) and a chloroform:isoamylalcohol (24:1) extraction was performed. The pH of the aqueous solution was adjusted to about 5 with acidic acid and nucleic acids were precipitated by addition of 0.6 volume isopropanol and 0.05 volume 4 M NaCl and incubation for 2 hrs at -20°C. After centrifugation (20–30 min., 10000 *g *at 4°C) the nucleic acids containing pellet was resuspended in 10 ml H_2_O_DEPC _containing 0.1% SDS. Total RNA was precipitated by addition of 0.25 volume 8 M LiCl and incubation for at least 15 hrs at 4°C with subsequent centrifugation for 20 min at 4°C, 10000 *g*. Total RNA was resuspended in 400 μl H_2_O_DEPC_. After ethanol precipitation (addition of 0.1 volume sodium acetate, 3 M pH4.8, and 2.5 volume ethanol) total RNA was resuspended in a volume of 50–100 μl H_2_O_DEPC_. The isolation of poly(A)+ RNA was carried out with the Oligotex Kit according to the manufacturers protocol (Qiagen; Hilden, Germany). Measurements of RNA yield and electrophoretic separation on formaldehyde gels were done following standard protocols [[32], modified].

Genomic DNA was isolated from sugar beet genotypes 1K0088 and 4B5421 according to a previous published method [33]. For recombinant DNA work standard techniques were employed [32].

### Suppression subtractive hybridization

The synthesis of cDNA was performed using the CLONTECH PCR-Select™ cDNA Subtraction Kit (Heidelberg, Germany). Each synthesis was carried out with 8 μg poly(A)+ RNA from sugar beet isolated either from leaves or taproots. Subtractive hybridization was done following the user manual (PT1117-1) of the CLONTECH PCR-Select™ cDNA Subtraction Kit. After the second PCR the amplified fragments from the forward and the reverse subtraction were cloned into the PCR cloning vector pCR^®^2.1. For each microgram PCR product approximately 300 recombinant plasmids were obtained. For the cloning of PCR products the Invitrogen T/A Cloning^® ^Kit was employed (Karlsruhe, Germany). Prior to ligation into pCR^®^2.1 the subtracted PCR cDNA products were subjected to an additional incubation of 1 hour at 72°C with dATP and *Taq *polymerase (TaKaRa; Gennevilliers, France) to ensure that the majority of the PCR fragments contain "A-overhangs" for an efficient cloning into the T/A cloning vector.

### DNA sequence analysis

The inserts of the plasmids were sequenced with fluorescently labeled M13 reverse and forward (-20) primers using the AutoRead Sequencing Kit (Pharmacia) and the Automated Laser Fluorescent A.L.F.™ DNA Sequencer from Pharmacia LKB (Freiburg, Germany). The DNA sequence analysis of the genomic and full-length cDNA clones was done by the custom sequencing service of MWG Biotech AG (Ebersberg, Germany). Sequences were subjected to data bank analysis using the BLAST algorithms [34] and analysed with the PILEUP programme of the GCG Wisconsin Analysis Package. For further promoter analysis the TRANSFAC^® ^database was employed [11]. DNA sequences were also processed and analysed on a Macintosh computer using DNA Strider 1.3 [35] and a PC computer using Vector NTI Suite 8.0 (Informax).

### Southern and Northern blot hybridizations

Radioactive probes were generated by the method of random hexamer priming with the Amersham Multiprime DNA Labelling System (Freiburg, Germany). Southern and Northern hybridizations were carried out following standard protocols [32,36].

For genomic Southern blot hybridizations 10 μg of DNA from sugar beet genotypes 1K0088 and 4B5421 was digested with different restriction enzymes. Electrophoretic separation, transfer to Hybond nylon membranes (Amersham Pharmacia Biotech, Freiburg), hybridization to radioactive probes, and exposure of the membrane to X-ray films were done according to standard protocols [32]. Radioactive probes were generated by labelling 20 ng of DNA with 50 μCi P^32^-dATP (6000 Ci/mMol, Amersham Pharmacia Biotech, Freiburg).

### Isolation of cDNA and genomic clones

A leaf specific, directional cDNA library from sugar beet genotype 4B5421 was synthesized by the custom cDNA library service of GIBCO BRL (Rockville, USA) and cloned into the plasmid vector pCMV Sport 6.0. Screening of the library was done according to standard protocols [32]. Seven positive *cab *cDNA clones were identified after screening of 10000 clones using the SSH fragment L2 as a probe (Table 1). The longest cDNA is 1062 bp long, harbors a 114 bp non-translated leader, a 756 bp long reading frame, a 177 bp 3' nontranslated leader, and a 15 bp poly A tail (data not shown, GenBank Acc. Nr. AJ579711).

A genomic library from sugar beet genotype 1K0088 was generated in the lambda vector EMBL3 SP6/T7 and screened using standard protocols [32]. Genomic clones for two different *cab *loci were isolated.

The promoter for the gene *cab*11 is present on a *Cla*I fragment that was subcloned into a plasmid vector and completely sequenced. The fragment is 6294 bp long and contains 51 bp from the coding region of the gene. The resulting plasmid was designated pC1a. Additionally, a 6026 bp large *Sal*I/*Cla*I fragment was released from the phage clone and subcloned into a Bluescript plasmid and designated pC1b. The promoter for the gene *cab*12 is present on a *Pst*I fragment that was also subcloned into a plasmid vector and completely sequenced. The fragment is 4002 bp long and the harboring plasmid was designated pC2.

From both genomic clones 1148 and 3049 base pairs containing most of the upstream region were deposited in GenBank (Acc. Nr. AX449166 and AX449167). The 1148 bp promoter fragment is designated *cab*11 promoter and the 3049 bp fragment *cab*12 promoter.

### Promoter reporter gene constructs

For transient gene expression assays, promoter fragments were linked as translational fusions to the luciferase reporter gene from *Photinus pyralis *in the reporter gene vector pGEM-luc (Promega, Mannheim). To introduce a plant polyA addition signal into pGEM-luc the respective fragment was isolated from pBI101.3 (Clontech, Heidelberg) by *Eco*RI digestion, followed by a Klenow fill in reaction and by redigestion with *Sac*I. This released a 260 bp DNA fragment from the nopaline synthase (*nos*) gene containing the polyA addition signal. To directionally clone this fragment into pGEM-luc, this plasmid was first linearised with *Sfi*I, treated with T4-polymerase to generate blunt ends and subsequently redigested with *Sac*I. After inserting the *nos *fragment the resulting plasmid was designated pLuc-nos2. To insert the *cab*11 promoter fragment, a *Sal*I(fill in)-*Avi*II fragment was cloned into the *Apa*I linearised and T4-polymerase treated plasmid pLuc-nos2. This plasmid harbors 1145 bp from the *cab*11 promoter including the coding sequence for the first 16 amino acids of the *cab*11 gene. This plasmid was designated pC1L-1097. In this plasmid the luciferase gene is translationally fused with the first 16 amino acids from the *cab*11 gene. A second plasmid was generated which harbors additional upstream sequences. Towards these ends a 6099 bp *Kpn*I fragment was released from the plasmid pC1b (see above) and the ends treated with T4-polymerase. The fragment was redigested with *Not*I and the desired fragment was directionally cloned as a *Kpn*I(blunt end)-*Not*I fragment upstream of the *cab*11 fragment in pC1L-1097. To generate compatible ends pC1L-1097 was digested with *Hin*dIII treated with T4-polymerase and redigested with NotI. The resulting plasmid was designated pC1L-7126.

To clone the promoter for gene *cab*12 upstream to the luciferase coding region, the promoter fragment from pC2 was released by *Not*I/*Eco*RI digestion and subsequently subjected to a partial digestion with *Avi*II. A 3100 bp long *Not*I/*Avi*II fragment was purified and subcloned into pLuc-nos2. The plasmid pLuc-nos2 was digested with *Apa*I, the ends treated with T4-polymerase and redigested with *Not*I. After ligation the resulting plasmid was designated pC2L-2998. In this plasmid the luciferase gene is translationally fused with the first 16 amino acids from the *cab*12 gene. To generate 5' promoter deletions pC2L-2998 was (1) digested with *Kpn*I/*Not*I, T4-polymerase treated, and religated to yield pC2L-1827, (2) digested with *Sma*I and religated to yield pC2L-989, and (3) digested with *Not*I and *Sal*I (partial), Klenow polymerase treated, and religated to yield pC2L-342.

For stable transformation the *cab*11 and *cab*12 promoters were cloned 5' to the β-glucuronidase gene (*uid*A). Towards these ends a 1.17 kb *Hin*dIII/*Bam*HI fragment was released from pC1L-1097 and cloned into the binary vector pBI101.3 (Clontech, Heidelberg). The resulting plasmid pC1G-1097 harbors a translational fusion between the first 16 amino acids of the *cab*11 gene and the *uid*A gene. Similarly, the *cab*12 promoter was released as a *Pst*I fragment from plamid pC2L-2998, treated with T4-polymerase then digested with *Bam*HI and subcloned into pBI101.3 which was linearised with *Sal*I, ends filled in with Klenow and redigested with *Bam*HI. The resulting plasmid was named pC2G-2998.

### Transient and stable gene expression assays

The luciferase expression from plasmids pC1L-1097, pC1L-7126, pC2L-2998, pC2L-1827, pC2L-989, and pC2L-342 were measured in sugar beet leaves after biolistic transformation [37]. For biolistic transformation the PDS-1000/He Particle Delivery System (BioRad, München, Germany) was used. Microcarrier was gold powder type 200-03 (Heraeus, Hanau, Germany) with a diameter of 1.09–2.04 micrometer. The transformation protocol supplied by the manufacturer of the particle delivery system was followed. Equimolar amounts of plamids pC1L-1097 and pC1L-7126 were used. Similarly, equimolar amounts of plamids pC2L-2998, pC2L-1827, pC2L-989, and pC2L-342 were used. To quantify gene expression the transformation control plasmid p70Sruc harboring the luciferase gene from *Renilla reniformis *under the control of the doupled CaMV 35S promoter was employed as a second reporter gene [38]. For each reporter gene construct three (pC1L-series) or four (pC2L-series) bombardments were made, gene expression strength of both luciferases measured and normalised relative to the luciferase expression of p70Sruc (see below). For each bombardment 13 leaf discs of equal diameter were cut out of sugar beet leaves and preincubated for 6 hours in petri dishes on MS-media containing 0.4 M mannitol at 25°C. The particle delivery conditions were 1550 psi, 9 cm distance and 27 Hg low pressure. After bombardment the petri dishes with the leaf discs were incubated for 16 h at 25°C under constant light. The *Photinus *and *Renilla *luciferase activity were measured with the dual-luciferase reporter assay system (Promega, Mannheim, Germany) in a Lumat 9501 luminometer (PE Biosystem) according to the protocol of the supplier.

For the generation of transgenic plants pC1G-1097 and pC2G-2998 were directly transformed into *Agrobacterium tumefaciens *strain GV2260 [39]. *Agrobacterium tumefaciens *mediated transformation techniques were performed with the binary T-DNA plasmids on sugar beet (*Beta vulgaris*, var. VRB) according to [40]. Selection of the transgenic plants was carried out on kanamycin. β-Glucuronidase (GUS) activity in crude leaf extracts was determined as described by Jefferson et al. [41] using 4-methylumbelliferone beta-glucuronide as a substrate. The concentration of the product 4-methylumbelliferone (Mu) was determined with a multiwell fluorescence plate reader (Millipore CytoFluor 2350). Protein content was measured by the method of Bradford (BioRad protein assay kit). Enzyme activity was calculated as pmol Mu × min^-1 ^× mg^-1^.

## Authors' contributions

DS isolated the cDNA and genomic clones, analysed the transcription during different developmental stages and tissues, generated promoter reporter gene constructs, transformed sugar beet, and performed the quantitative reporter gene assays. DUK performed the suppression subtractive hybridization and analysed the homology and tissue specificity of the cDNA fragments. RH identified *cis*-regulatory elements. RH and DS conceived of the study, and participated in its design and coordination.

## Figures and Tables

**Table 1:** Homology of cDNA fragments enriched for leaf expressed genes.

cDNA-fragment	Base pairs	Nearest homolog^a^
L1	862	*Flaveria pringlei *mRNA for glycine hydroxymethyltransferase (371 bp, 84 %) Acc. Z25859; [42]
L2	479	*Nicotiana tabacum *mRNA for light-harvesting Chl a/b binding protein (337 bp, 86 %) Acc. X82497
L3	424	*Spinacia oleracea *psaH mRNA for photosystem I reaction centre subunit VI (361 bp, 90 %) Acc. X16858 [43]
L4	508	unknown
L5	587	*Spinacia oleraceae *ALDCHL mRNA for fructose-1,6-bisphosphate aldolase (495 bp, 87 %) Acc. X66814 [44]
L6	383	*Beta vulgaris *clone RUB109UNI ribulose bisphosphate carboxylase, small subunit gene, partial sequence (139 bp, 98 %) Acc. AF173667 [45]
L7	451	unknown
L8	221	*Arabidopsis thaliana *mRNA for hydroxypyruvate reductase, complete cds (203 bp, 83 %) Acc. D85339
L9	520	*Spinacia oleracea*, ferredoxin-thioredoxin reductase A2 mRNA (151 bp, 82 %) Acc. X77163 [46]
L10	643	unknown
L11	338	*Beta vulgaris *clone RUB109UNI ribulose bisphosphate carboxylase, small subunit gene, partial sequence (206 bp, 93 %) Acc. AF173667 [45]
L12	532	unknown
L13	265	*Spinacia oleraceae *rubisco activase mRNA, complete cds (256 bp, 89 %) Acc. J03610 [47]
L14	540	*Spinacia oleraceae *psaL mRNA for subunit XI of photosystem I reaction center (409 bp, 88 %) Acc. X64445 [48]

^a^The genes that gave the highest homology in a BLASTN gene bank comparison are listed. In parenthesis, the contiguous stretch of base pairs showing the indicated percent homology is given.

**Table 2:** Transient gene expression analysis of two CAB promoters in sugar beet leaves. The plasmids harbor promoter fragments of *cab*11 (pC1L) and *cab*12 (pC2L).

Promoter Reporter Gene Construct^a^	Relative expression level in sugar beet leaves^b^
pC1L-7126	7.0 +/- 3.4^c^
pC1L-1097	9.6 +/- 4.6
pC2L-2998	8.0 +/- 0.2
pC2L-1827	4.5 +/- 0
pC2L-989	6.4 +/- 2.4
pC2L-342	6.5 +/- 1.9
CaMV 35S	30.2 +/- 16.6

^a^The number following the identifier of the promoter relates to fragment length

^b^The relative expression level was calculated as:

(*Photinus *value_(construct) _- *Photinus *value _(without DNA) _/ *Renilla *value _(construct) _- *Renilla *value _(without DNA)_) × 100.

^c^Average of at least three assays +/- standard deviation

**Table 3:** Induction of two *cab *promoters seven days after illumination of etiolated sugar beet plants. The results for two *cab*11 (pC1) and two *cab*12 (pC2) promoter lines are shown.

	Experiment 1			Experiment 2		
	
	Etiolated leaves, specific activity^a^	Green leaves, specific activity	Light induction	Etiolated leaves, specific activity	Green leaves, specific activity	Light induction
non- transgenic	10	0	0	16	45	2,8
C1-121	155	1287	8,3	42	4126	98
C1-122	36	153	4,3	50	5611	112
C2-50	30	181	6	44	5207	118,4
C2-52	n.d^b^	n.d	n.d	43	4107	95,5

^a^pmol Mu × min^-1 ^× mg^-1^

^b^not determined
